# c-Met expression and activity in urogenital cancers – novel aspects of signal transduction and medical implications

**DOI:** 10.1186/s12964-017-0165-2

**Published:** 2017-02-17

**Authors:** Ralf Hass, Susanne Jennek, Yuanyuan Yang, Karlheinz Friedrich

**Affiliations:** 1 Biochemistry and Tumor Biology Lab, Department of Gynecology, Hannover Medical School, Hannover, Germany; 2 Institute of Biochemistry II, University Hospital Jena, Nonnenplan 2-4, D-07743 Jena, Germany

**Keywords:** c-Met, HGF/SF signalling, c-Met inhibitors, MSC, Bladder, prostate and ovarian cancer

## Abstract

C-Met is a receptor tyrosine kinase with multiple functions throughout embryonic development, organogenesis and wound healing and is expressed in various epithelia. The ligand of c-Met is Hepatocyte Growth Factor (HGF) which is secreted among others by mesenchymal stroma/stem (MSC) cells.

Physiological c-Met functions are centred around processes that underly cellular motility and invasive growth. Aberrant c-Met expression and activity is observed in numerous cancers and makes major contributions to cell malignancy. Importantly, HGF/c-Met signaling is crucial in the context of communication between cancer cells and the the tumor stroma.

Here, we review recent findings on roles of dysregulated c-Met in urogenital tumors such as cancers of the urinary bladder, prostate, and ovary. We put emphasis on novel aspects of cancer-associated c-Met expression regulation on both, HGF-dependent and HGF-independent non-canonical mechanisms. Moreover, this review focusses on c-Met-triggered signalling with potential relevance for urogenital oncogenesis, and on strategies to specifically inhibit c-Met activity.

## Background

**Fig. 1:**
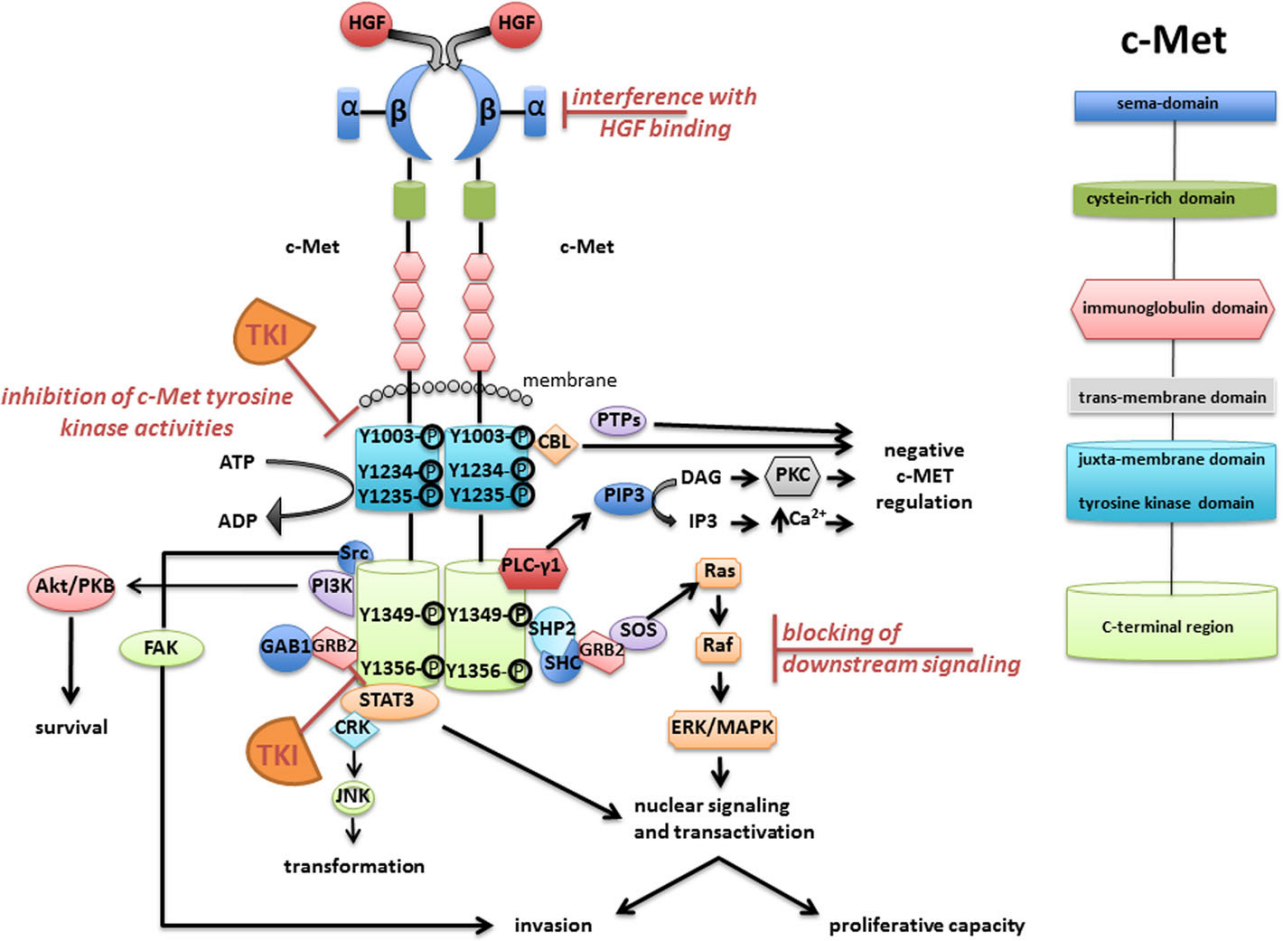
HGF/SF-mediated activation of c-Met and relayed downstream signalling. The c-Met receptor can be structured into distinct domains, including sema, cysteine-rich, immunoglobulin, trans-membrane, juxta-membrane, tyrosine kinase, and C-terminal region. Pharmacological intervention with activated c-Met signalling includes: (i) competitive interference with HGF/c-Met interaction, (ii) inhibition of the tyrosine kinase activity of c-Met with the use of tyrosine kinase inhibitors (TKI), or (iii) blocking of activated c-Met downstream signaling mediators. Accordingly, cell fate and development such as survival, transformation, cell motility, and proliferative capacity can be affected. This figure was adapted from Organ and Tsao, 2011 [2]

**Fig. 2:**
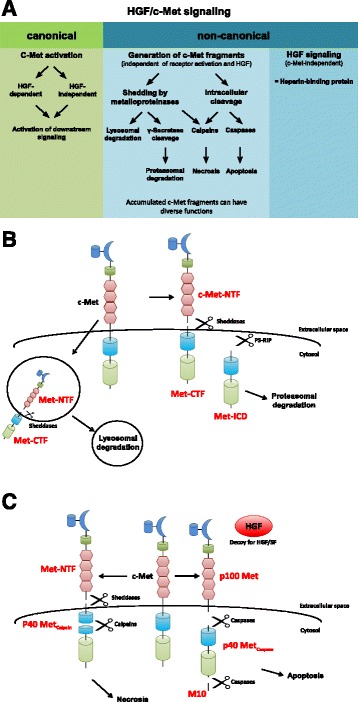
Pathways of c-Met signaling. **a** Overview of HGF/c-Met signaling via the canonical and non-canonical pathway. Canonical or “classical” HGF/Met signaling involves ligand-dependent and independent receptor activation which leads to the induction of downstream signaling cascades (*left*). Non-canonical HGF/c-Met signaling is independent of receptor activation. Generation of c-Met receptor fragments takes place under various cellular conditions such as apoptotic and necrotic stimuli as well as in the context of specific physiological circumstances. HGF is also able to exert signals independently of c-Met, e.g. upon interactions triggered by its heparin-binding domain. **b** Generation of c-Met fragments via shedding and cleavage by γ-secretase: Sheddases or metalloproteinases cleave full-length c-Met within its extracellular domain, resulting in different in a soluble extracellular N-terminal fragment (Met-NTF) and a membrane-associated C-terminal fragment (Met-CTF). Met-CTF can be further processed by the γ-secretase complex by presenilin-dependent intramembrane proteolysis (PS-RIP) into an intracellular domain (Met-ICD) which is routed to proteasomal degradation. Full-length membranous c-Met can also be internalized and cleaved by sheddases giving rise to Met-NTF and Met-CTF. These intracellularly generated c-Met fragments undergo lysosomal instead of proteasomal degradation. **c** Origin of c-Met fragments through intracellular cleavage by caspases and calpains: In response to apoptotic stimuli, c-Met is cleaved at two distinct sites in the intracellular domain by activated caspase-3, resulting in membrane-anchored p100 Met, a 40 kDa cytosolic p40 Met fragment and a small peptide (M10). Under necrotic conditions, c-Met is cleaved by metalloproteinases and further processed by calcium-independent proteases (calpains) instead of γ-secretase. The resulting product p40 Metcalpain differs from p40 Metcaspase by a few amino acid residues and is not able to promote apoptosis

c-Met (mesenchymal epithelial transition factor) is a multifunctional transmembrane tyrosine kinase and acts as a receptor for hepatocyte growth factor/Scatter factor (HGF/SF) [1]. It is expressed in various epithelial tissues (liver, pancreas, prostate, kidney, muscle, bone-marrow) during embryogenesis [2] and is also found on the cell surface of numerous tumorous cell populations. Shortly after its discovery, multiple oncogenetic properties of c-Met were described, including the stimulation of cell dissociation, migration, motility, and invasion of extracellular matrix [3–6]. Formation of mature c-Met is achieved by proteolytic cleavage of a precursor in a post-Golgi compartment, resulting in a small alpha and large beta polypeptide which then associate into a heterodimer. A disulfide bridge connects the small alpha unit and the extracellular segment of the membrane spanning beta subunit [7]. The extracellular part of the beta subunit is composed of an N-terminal sematophorin (sema) domain (essential for receptor activation) followed by a cysteine-rich portion (plexin sematophorin domain) and four IPT (immunoglobulin like plexins transcription factor) domains. A transmembrane helix connects the extracellular domain of c-Met to its intracellular section which can be divided into a juxtamembrane domain, a tyrosine kinase domain and the C-terminal region [2] (Figs. 1 and 2).

### Structure and function of c-Met

C-Met becomes activated through homo-dimerisation upon binding of its ligand HGF [8–12]. As a result of ligand-induced dimerization, the intracellular tyrosine kinase domains of the two receptor beta-subunits trans-phosphorylate each other at residues Tyr^1234^ and Tyr^1235^ within the catalytic loops [13]. This event fully unleashes the tyrosine kinases which subsequently catalyze further phosphorylation of the receptor chain at Tyr^1003^ in the juxtamembrane domain and Tyr^1349^/Tyr^1356^ within the C-terminal region (Fig. 1). These modifications relay further signals by generating specific contact interfaces (pTyr-containing motifs) with a variety of different src homology domain 2 (SH2) containing adaptor molecules to eventually regulate induction of cell motility, proliferation, invasiveness, and tubular morphogenesis [14–22].

C-Met receptor downstream signaling is mediated by recruitment of distinct adaptor and effector proteins such as src-homology-2- (SH2-), src-homology-3- (SH3-) domain-containing proteins and phospho-tyrosine-binding proteins (PTB) (Fig. 1). These adaptor proteins without enzymatic activity include Grb2 (growth factor receptor-bound protein 2), CRK ((CT10 (chicken tumor virus number 10) regulator of kinase)), and GAB1 (GRB2-associated binding protein 1). C-Met-binding proteins with enzymatic activity include Src kinases, PI3K (phosphatidylinositol 3-kinase), PLC-γ1 (phospholipase C-γ1), SHIP-2 (SH2 domain-containing 5’ inositol phosphatase), STAT-3 (signal transducer and activator of transcription), SOS (Ras guanine nucleotide exchange factor son-of-seven), PTPs (protein tyrosine phosphatases), e.g. SHP2 (SH2 domain-containing phosphatase-2) [14, 16–23]. HGF-stimulated c-Met activation can also phosphorylate downstream targets such as p44/42 MAP kinase (ERK) to mediate enhanced proliferation and cell cycle progression [2, 24, 25] (Fig. 1). In addition, Met can be activated even in the absence of HGF. In this context, Fan et al. described a novel HGF-indepedent mechanism in which non-receptor tyrosine kinase FER is involved. The upregulation of FER in ovarian cancers could trigger a signaling cascade downstream of c-Met in a HGF independent manner. Mechanistically, FER was shown to phosphorylate the tyrosine residue of the C-terminal SH2 domain docking site of c-Met, thus maintaining recruitment of signaling molecules such as GAB1 and Grb2 to the receptor. Most importantly, FER-mediated downstream signalling by c-Met could not be inhibited by a c-Met inhibitor alone [26].

### Intra- and intercellular signaling triggered by c-Met in urogenital cancers

Activated c-Met in normal cells – predominantly in stromal cells including MSC - plays a major role in organogenesis and wound healing whereas in tumor cells, HGF-activated c-Met contributes to invasive and metastatic behavior [12, 27]. Various urogenital cancers like muscular-invasive urinary bladder cancer, prostate cancer and ovarian cancer express c-Met [28–30]. c-Met can be detected in about 70% of the different types of ovarian cancers such as low and high grade serous, endometrioid, clear cell and mucinous carcinomas, in about 30% of the tumors c-Met is expressed to a noticeably high degree [31–33]. C-Met over-expression is observed particularly in hyperactive tumors which also show elevated angiogenesis and neo-vascularization due to activity of vascular endothelial growth factor receptor-2 (VEGF-R2). These tumor properties correlate with poor prognosis [34–36].

In prostate cancer, it was shown that androgen deprivation is connected with a more aggressive phenotype and leads to an increased c-Met expression. In parallel, the androgen receptor, which is known as a negative regulator of c-Met, is downregulated [37–39]. Hence, c-Met signaling obviously has an important role in maintaining survival as well as proliferation in androgen receptor-independent prostate cancer cells [40]. In this context, evidence has been obtained for an association of c-Met activation with decreased expression of genes related to DNA repair. It was, thus, suggested that c-Met may contribute to the accumulation of DNA damage and mutations and consequently favor progression of castration-resistant prostate cancer [40].

A recent report shows that c-Met upregulation in castration-resistant prostate cancer tissue is associated with a different distribution pattern compared to biopsies from naïve prostate cancer tissue. Interestingly, a c-Met fragment lacking the c-terminal part of full-length membranous c-Met was found to accumulate in the nuclei of tumor cells preferentially in castration-resistant prostate cancer [41]. These results support the notion of a role for full-length and processed c-Met in the progression of androgen-independent prostate cancer.

Elevated c-Met expression in tumor cells also leads to enhanced c-Met activity within the tumor tissue, since profound HGF expression within the tumor stroma activates c-Met on cancer cells in a paracrine manner. HGF expression was shown to be clearly increased in urogenital cancers such as urinary bladder, prostate and ovarian cancer [42–45]. In prostate and bladder cancer, c-Met-related feedback activation crosstalk between carcinoma cells and tumor-associated fibroblasts was characterized: HGF secretion becomes upregulated in stromal fibroblasts co-cultured with tumor cells, which in turn enhances migratory and invasive properties of cancer cells [44, 46–51]. However, in normal ovary HGF is mainly detected in the ovarian surface epithelium on the outer surface of the ovary and to a lesser extend in ovarian stromal cells directly adjacent to the ovarian surface epithelium [45]. Moreover, an elevated HGF expression is detectable in epithelial cell components of ovarian tumors and in MSC suggesting an autocrine and/or paracrine stimulation of ovarian tumor growth including tumor cell protection [45, 52–54]. In other cancer entities such as gastric and colon cancer, HGF/c-Met signaling was shown to be operative in fibroblasts within the tumor microenvironment, thereby contributing to tumor progression [55, 56]. Moreover, immunohistochemical analysis showed c-Met expression on myofibroblasts mainly in the invasive area of lung adenocarcinoma [57]. c-Met expression on myofibroblasts in patients with small-sized lung adenocarcinoma was correlated with shortened patient survival [57]. It will be important to address in how far HGF-mediated fibroblast activation does also play a role in urogenital cancers.

### Non-canonical signaling by c-Met

It is now well established that receptor tyrosine kinases can also act via signaling pathways other than the Ras/Raf cascade. “Canonical signaling” is currently used as a term for the signaling route extending from ligand binding to the cell surface exodomains of receptors resulting in dimerization/oligomerisation and receptor trans-/ autophosphorylation with subsequent intracellular signal release. Recently, “non-canonical signaling” has emerged as a concept to describe the specific generation of defined intracellular tyrosine kinase receptor fragments which have different functions [58]. The formation of these fragments is achieved by the activity of different proteases whose functions can be dependent or independent of receptor stimulation by ligand (Fig. 2).

Four cleavage products of c-Met with potential functions in signaling have as yet been described. The extracellular N-terminal fragment (Met-NTF) can be eliminated from the full-length receptor by metalloproteinases such as ADAM-10 and ADAM-17. Subsequent processing of the membrane-proximal C-terminal fragment (Met-CTF) by presenilin-dependent, regulated, intramembrane proteolysis (PS-RIP) results in an intracellular c-Met fragment (Met-ICD) which becomes subject to proteasomal degradation [59–62]. It is controversal as to whether the extracellular soluble domain of c-Met (NTF) exerts the physiological function of sequestering and, thus, antagonizing HGF/SF and is also a potent antagonist to the antagonist [63–65]. However, in mouse xenograft experiments with injected UOK261 bladder carcinoma cells, which show a high proteolytic cleavage rate of the full-length receptor, a linear relationship between soluble c-Met in the plasma or urine and total tumor volume was shown [63, 64]. Moreover, the level of soluble c-Met in urine from bladder cancer patients was shown to be a disease marker and to even allow for distinction between tumor stages as muscle no-invasive and invasive cancers [66]. These findings suggest that cleavage of c-Met plays a role in urinary bladder cancer progression.

By shedding the Met-NTF, the resulting fragments Met-NTF and-CTF can also be lysosomally degraded. Ancot et al. published a model describing the fate and subcellular location of the receptor-derived breakdown products post processing of the full-length cell surface receptor by PS-RIP and its proteasomal degradation upon removal of Met-NTF. However, if c-Met is membrane-bound and internalized, it can also undergo intracellular proteolytic cleavage. This process gives rise to Met-NTF and Met-CTF, which can subsequently be degraded lysosomally. Thus, fragment Met-CTF escapes processing by PS-RIP when the cleavage takes place intracellularly [67]. Both shedding-induced degradation pathways are independent of receptor activation and may serve the function of preventing overexpression and "over-activation" of c-Met [60, 61, 67].

Experimental overexpression of a segment comprising only the intracellular segment of c-Met devoid of the extracellular domain in NIH3 Ts fibroblasts showed an elevated oncogenic potential with enhanced proliferation and invasiveness compared to control fibroblasts [68]. Interestingly, constitutively active c-Met fragments were detected in cell nuclei of an aggressive breast carcinoma cell line. They showed trans-activating activity in an experimental Gal4-based transactivation assay and appear to have a function in connection with spontaneous migratory behaviour of this cell line [69]. However, the absence of tumor suppressor Wwox plays a role here. Wwox is believed to possess regulatory functions in both controlling the stability of full-length c-Met and in the inhibition of the transactivation function of nuclear Met-CTF [69]. As already mentioned above, the nuclear Met-ICD level is higher in castration-resistant than in naïve prostate cancer. Withdrawal of androgens upregulates nuclear Met-ICD which in turn activates transcription factor SOX9 and the β-catenin/ androgen receptor signaling pathway. In combination, this promotes cell transformation and three-dimensional tumor growth in castration-resistant prostate cancer [41]. These results suggest that proteolytically generated c-Met fragments that are not lysosomally or proteasomally degraded and thus, accumulate in the cell which can contribute to tumor progression. However, as yet little is known about the precise physiological or pathological mode of action and function of these fragments.

c-Met cleavage products are functional in the control of cell death and survival [70]. The receptor can be cleaved intracellularly at two distinct sites in response to stress stimuli. The resulting 40 kD cytosolic p40Met fragment, in contrast to the ligand-activated full-length receptor, acts pro-apoptotically via the mitochondrial permeabilization process of the intrinsic apoptotic pathway. Caspase-3 was identified as the responsible protease for the cleavage of c-Met [71–73].

Cleavage of c-Met yields the intracellular p40Met fragment, a membrane-bound p100Met fragment and, in addition, a peptide ten amino acid residues in length designated M10 and corresponding to C-terminal end of the receptor protein [74, 75]. The p100Met fragment encompassing the entire extracellular domain, is still membrane-bound and can act as a scavenger of HGF/SF, thereby serving as an inhibitor of the HGF/c-MET signal cascade [74]. M10 can block TGF-β-induced SMAD2 phosphorylation by interaction with SMAD2 and, thus, exerts antifibrotic effects [75].

The ratio of full-length c-Met and caspase-3 appears to have an influence on the extent of apoptosis in hepatocytes: High abundance of c-Met can suppress caspase-3 activity and, thus, suppress apoptosis. Under conditions of a strong apoptotic stimulus, however, this inhibitory effect of c-Met on caspase-3 is overcome and cell death is no longer blocked [76].

Remarkably, caspase-3 is also active in non-apoptotic melanoma and glioblastoma cells as well as in invasive bladder cancer promoting tumor progression [77–79]. Mukai et al. published that cancer cells are able to react on apoptotic stimuli with a higher invasiveness through caspase activation [80]. Until now there is no evidence for a role of caspase-3 mediated cleavage of c-Met in this context. Moreover, c-Met also becomes cleaved in necrosis [81] whereby c-Met can be shed by metalloproteases as discussed above and instead of further processing by the γ-secretase complex, it becomes degraded by calcium-dependent proteases (calpains). This process gives rise to a 40 kDa Met fragment designated p40Met calpain which differs from the previously described p40Met caspase fragment in a few amino acids and its inability to promote apoptosis [81]. Moreover, the mutation R970C found in lung cancer within the juxtamembrane domain of c-Met promotes the calpain-dependent formation of a novel 45 kDa fragment p45Met, particularly in confluent cells [82]. Overexpression of p45Met in epithelial cells enhances HGF-mediated cell scattering and invasion without induction of tyrosine kinase activity of p45Met. This indicates an alternative mechanism besides activation of cell scattering by full length receptor [82]. In addition, ectopic expression of R970C variant of Met in the suspension cell line Ba/F3 did not show any transforming capacity, supporting that HGF treatment, proteolytic cleavage and cell confluence are probably necessary for cancer-related effects of this variant [83].

Analysis of Met-overexpressing tumor samples from patients with non-small cell lung cancer demonstrated the existence of all Met fragments suggesting a variety of cellular conditions within lung tumors [81]. However, little is known about the role of the different c-MET fragments in tumor progression so far.

Interestingly, not only c-Met can contribute to tumor progression in a ligand-independent manner. Tate et al. indicated a role for c-Met ligand HGF in prostate cancer in the absence of its receptor [84]. HGF induces cell adhesion and migration in a c-Met-negative prostate cancer cell line on the basis of a low-affinity interaction between its heparin-binding domain and nucleolin. Nucleolin is a nuclear protein which is known to localize also to the surface of cells. In this setting, it may link HGF and integrin function [84]. Low or high concentrations of HGF have been observed to exert distinct functions also on non-tumorigenic cells, leading to the postulation that HGF can elicit cellular effects in a c-Met-independent manner in parallel with or alternatively to “classical” c-Met/HGF signaling [85, 86].

### Experimental and pharmacological interference with c-Met activity in urogenital cancer cells

The identification of prominent c-Met expression in certain urogenital tumors suggested that the HGF/SF – c-Met axis may serve as an attractive target to be included into chemotherapeutic regimens. Different approaches have been taken towards molecularly targeting c-Met and HGF such as (i) competitive interference with HGF/c-Met interaction, (ii) inhibition of the tyrosine kinase activity of c-Met, or (iii) blocking of activated c-Met downstream signaling mediators (Fig. 1). Accordingly, several preclinical studies indicated that inhibition of c-Met represents a promising therapeutic strategy paralleled by findings that c-Met overexpression has prognostic value in urogenital cancers, particularly in ovarian cancer.

Using an ovarian cancer mouse model, the orally available c-Met inhibitor PF-2341066 was successfully applied to reduce cell proliferation, adhesion and invasion as well as to induce apoptosis [87]. This small-molecule inhibitor shows specificity against c-Met and anaplastic lymphoma kinase (ALK) and reduced mouse tumor size paralleled by increased animal survival [88].

Another c-Met inhibitor, Crizotinib (PF-02341066), represents a designed tyrosine kinase inhibitor based on the core structure of the c-Met-inhibitor PHA 665752 in which some functional groups of the originating molecule (PHA 665752) were altered. As a result Crizotinib has stronger affinity for the ATP-binding pocket of the kinase domain within the c-Met receptor. Consequently, binding of ATP to the c-Met kinase domain and subsequent tyrosine phosphorylation of the receptor and signal mediators are blocked [89, 90]. Moreover, Crizotinib displays further inhibitory potential for the tyrosine kinase receptor ALK and ROS1 (ROS proto-oncogene 1, receptor tyrosine kinase) [91]. Crizotinib has, hence, been approved for the treatment of ALK-positive non-small cell lung carcinoma in the United States since August 2011. Studies employing a combination of Crizitonib together with PF-05212384 or gedatolisib were performed to additionally inhibit the phosphatidyl inositol-3 kinase (PI3K)/Akt/mammalian target of rapamycin (mTOR) signaling pathway in the tumor cells. Results demonstrated superior anti-tumor effects of this combination compared to the individual agents. However, this combined treatment was also associated with the development of a gedatolisib-resistant subline, indicating increased expression of the multi-drug-resistant-1 gene [92].

The orally administrable tyrosine kinase inhibitor Foretinib (XL880, GSK1363089) exhibits a certain specificity and high affinity binding for both c-Met (IC_50_: 0.4 nM) and vascular epithelial growth factor receptor-2 (VEGF-R2/KDR) (IC_50_: 0.9 nM) [93]. This drug can also bind to a variety of other growth factor receptors, however, with significantly reduced binding affinities: PDGFRα (IC_50_: 3,6 nM), PDGFRβ (IC_50_: 9.6 nM), RON (IC_50_: 3 nM), c-KIT (IC_50_: 6,7 nM), FLT-3 (IC_50_: 3,6 nM) und TIE-2 (IC50: 1,1 nM) and low affinities to FGFR1 (IC50: 660 nM), und EGFR (IC_50_: 2,9 μM) [93–95]. An association of Foretinib with receptor tyrosine kinases is accompanied by a conformational change within the kinase structure and deprivation of enzymatic activity. Notably, Foretinib binding to its target tyrosine kinases receptors remains stable for at least 24 h [95].

Foretinib has also been demonstrated to block MAP kinase signaling and cell cycle progression during in vitro studies, substantiating this drug as a multi-target tyrosine kinase inhibitor [96]. Promising in vivo studies with significant Foretinib-mediated reduction of tumor size were performed in NSCLC [91], in lung metastases [93], and in Hedgehog medulloblastoma [97].

Potential effects of Foretinib as a multi-target tyrosine kinase inhibitor besides its specificity for c-Met are also focused on inhibition of VEGF-R2 signaling as an important pathway during angiogenesis and subsequent neo-vascularization. Here, Foretinib exhibits properties which primarily target the tumor microenvironment, thereby displaying dual anti-tumorigenic strategies. Promising effects of Foretinib in targeting VEGF-R2 signaling were achieved by an approximately 10-fold reduction in tumor size of tumors of the rare and aggressive small cell carcinoma of the ovary, hypercalcemic type (SCCOHT), following tumor initiation by the two model cell lines SCCOHT-1 and BIN-67, respectively. MSC within the tumor microenvironment were shown to contribute to this outcome [52, 53, 67, 98, 99]. Supportive evidence of Foretinib’s anti-tumor activity through inhibition of c-Met- and VEGFR-2-mediated signal transduction was also demonstrated in further tumor models e.g. hepatocellular carcinoma [100], kidney cell carcinoma [101] and gastric carcinoma [102].

The tumor microenvironment plays an pivotal role for therapeutic approaches [103–105]. The importance simultaneously targeting tumor cells and the tumor microenvironment of urogenital cancers by anti-cancer drugs in is also underscored by results with DCC-2701 as a c-Met/TIE-2/VEGF-R inhibitor [106]. In the course of stroma–cancer cell interactions, human ovarian fibroblasts and MSC produced and secreted HGF, leading to elevated growth and migration of ovarian cancer cells coinciding with c-Met phosphorylation at Tyr_1349_. Tumor treatment with DCC-2701 was able to efficiently reduce the tumor burden in vivo by inhibition of c-Met phosphorylation and c-Met-mediated signaling, for cell growth and migration [106].

## Conclusions

In summary, the abundance of c-Met expression and intracellular signaling in urogenital cancers may provide a selective molecular target for tumor therapeutic interventions. Moreover, multi-target c-Met tyrosine kinase inhibitors with the property to simultaneously affect cancer cells and the tumor microenvironment represent efficient and focused drugs to target urogenital cancers. In this context, it is important to note that cancer growth and metastasis of c-Met-positive urogenital cancers involves further stimuli besides HGF-binding and subsequent c-Met signaling since no anti-tumor effect was detectable in an ovarian cancer clinical trial after using a humanized IgG2 antibody directed against HGF (AMG-102), even though AMG-102 prevents HGF binding to c-Met and subsequent c-Met activation [107]. It is likely that the above-mentioned and not entirely understood non-canonical c-Met signaling mechanisms as well as c-Met-independent HGF activity are involved in this phenomenon.

## Abbreviations

c-Met: Mesenchymal epithelial transition factor
CRK: CT10 (chicken tumor virus number 10) regulator of kinase
GAB1: Grb2-associated binding protein 1
Grb2: Growth factor receptor-bound protein 2
HGF: Hepatocyte growth factor
IPT: Immunoglobulin-like plexins transcription factor
MAPK: Mitogen activated protein kinase
MSC: Mesenchymal stroma/stem cells
mTOR: Mammalian target of rapamycin
NFκB: Nuclear factor κB
PI3K: Phosphatidylinositol 3-kinase
PLC-γ1: Phospholipase C- γ1
PTB: Phospho-tyrosine-binding proteins
PTPs: Protein tyrosine phosphatases
semaphoring: Sema
SF: Scatter factor
SH2: Src homology domain-2
SH3: Src homology-3 domain
SHIP-2: SH2 domain-containing 5’ inositol phosphatase
SHP2: SH2 domain-containing phosphatase-2
SOS: Ras guanine nucleotide exchange factor son-of-seven
STAT-3: Signal transducer and activator of transcription
VEGF-R2: Vascular endothelial growth factor receptor-2
